# Orthopaedic Surgery Outcomes in Patients With Haemophilia A or B Treated With Extended Half‐Life Recombinant Factor VIII and IX Fc Fusion Proteins: A Multicentre Prospective Study

**DOI:** 10.1111/hae.70141

**Published:** 2025-10-10

**Authors:** Luigi Piero Solimeno, Roberta Gualtierotti, Emanuele Guido, Jacopo Acquati Lozej, Enrica Cristini, Alessandro Ciavarella, Sara Arcudi, Christian Carulli, Lisa Pieri, Simona Maria Siboni, Flora Peyvandi

**Affiliations:** ^1^ Division of Orthopaedic Surgery and Traumatology Fondazione IRCCS Ca' Granda Ospedale Maggiore Policlinico Milan Italy; ^2^ Angelo Bianchi Bonomi Hemophilia and Thrombosis Center Fondazione IRCCS Ca' Granda Ospedale Maggiore Policlinico Milan Italy; ^3^ Department of Pathophysiology and Transplantation Università degli Studi di Milano Milan Italy; ^4^ Department of Oncology and Hematology‐Oncology Università degli Studi di Milano Milan Italy; ^5^ Orthopaedic Clinic University of Florence Firenze Italy; ^6^ Center for Bleeding Disorders and Coagulation Department of Oncology Careggi University Hospital Florence Italy

**Keywords:** factor IX Fc fusion protein, factor VIII Fc fusion protein, haemophilia, joint diseases, orthopaedic surgery, total joint replacements

## Abstract

**Introduction:**

Haemophilia A and B are hereditary bleeding disorders that require multidisciplinary perioperative management. Data on orthopaedic surgery outcomes with extended‐half‐life (EHL) recombinant Fc‐fusion factor VIII (rFVIIIFc) and factor IX (rFIXFc) products remain limited.

**Aims:**

To evaluate the efficacy of EHL rFVIIIFc or rFIXFc in major orthopaedic surgery, focusing on haemostasis, safety and factor consumption.

**Methods:**

This prospective study involved persons with haemophilia A or B treated with rFVIIIFc or rFIXFc undergoing orthopaedic surgery.

**Results:**

Twenty major orthopaedic surgeries (2018–2023) were included in 19 persons with severe or moderate haemophilia A (*n* = 14) or B (*n* = 5), median age 46 years (range 26–60). Procedures included arthroplasty, arthrodesis, arthroscopic synovectomy, prosthetic revision of the knee or ankle, and removal of a femur fracture fixation device. Median hospital stay was 7 days (range 2–18). Median cumulative factor consumption was 300 and 388 IU/kg for haemophilia A and B, respectively. Haemostatic efficacy was rated as ‘good’ in 95% (*n* = 18) of cases, ‘poor’ in 5% (*n* = 1), and not documented in one case. Median haemoglobin (Hb) change was –2.0 g/dL (range –4.6 to +0.5); no transfusions were required. Complications were reported in 45% (*n* = 9) of cases (anaemia 40%; blood loss 5%) and managed with oral supplementation of iron and folates. No adverse events related to rFVIIIFc or rFIXFc administration were observed.

**Conclusion:**

RFVIIIFc and rFIXFc provide effective haemostasis during orthopaedic surgery in patients with haemophilia A and B, with a favourable safety profile. Further multicentre studies are warranted to confirm these results and refine perioperative management guidelines.

## 1 Introduction

Haemophilia A and B, caused by the deficiency of coagulation factor VIII (FVIII) and FIX, respectively [1], predispose individuals to spontaneous bleeding in the musculoskeletal system and consequent joint damage, often necessitating joint replacement [2].

According to the World Federation of Haemophilia (WFH) guidelines, a surgical procedure is considered major if it requires haemostatic support for periods exceeding 5 consecutive days [3]. In major orthopaedic surgery, an FVIII and FIX level of 80–100 IU/dL is recommended preoperatively and for at least 3 days after surgery [3].

The extended half‐life (EHL) Fc‐fusion products efmoroctocog alfa and eftrenonacog alfa (referred to herein as rFVIIIFc and rFIXFc, respectively), have contributed to improving haemophilia care by reducing infusion frequency and ameliorating haemostatic control [1, 4]. Molecular engineering has improved pharmacokinetics of EHL products thanks to a reduced proteolysis, decreased cellular endocytosis, and reduced renal and hepatic elimination, thus allowing less frequent dosing while reaching higher trough levels, compared to standard half‐life (SHL) drugs [5]. Despite their approval in Europe for replacement therapy, data on the perioperative use of rFVIIIFc and rFIXFc in the orthopaedic setting remain limited.

With this as background, the present study aims to provide clinical insights on the perioperative management of persons with haemophilia A and B undergoing major orthopaedic surgery with rFVIIIFc and rFIXFc replacement therapy by presenting the experience of two reference centres.

## 2 Materials and Methods

This multicentre, prospective study was conducted at the Angelo Bianchi Bonomi Haemophilia and Thrombosis Centre (Milan, Italy) and the Centro Traumatologico Ortopedico (Florence, Italy) between 2018 and 2023. The study was approved by Comitato Etico Milano Area 2 on 18th September 2018 (n°423_2018ter). Written informed consent was obtained from all participants prior to inclusion in the study. The study is reported in accordance with the Strengthening the Reporting of Observational Studies in Epidemiology (STROBE) guidelines [6].

Eligible participants included persons diagnosed with severe or moderate haemophilia A or B treated with rFVIIIFc or rFIXFc and undergoing major orthopaedic procedures.

Collected data included demographics, haemophilia type and severity, baseline prophylaxis regimen, joint health status and previous surgical interventions. Details on surgical procedures, including type, complexity, and duration, were obtained from clinical and surgical records.

Preoperative factor activity levels were assessed for all patients. Prior to surgery, a loading dose of rFVIIIFc or rFIXFc was administered to achieve recommended haemostatic targets in accordance with current guidelines [3]. Postoperative factor levels were systematically monitored based on clinical practice at predefined time points: at baseline and after factor administration (immediately prior to surgery), after surgery (within 0–2 h), at 24, 48 h, and at the time of hospital discharge, or at 1 week after surgery in the case of hospitalizations exceeding 7 days. This approach was necessary due to differences in patient management between the two centres: in Milan, patients were discharged earlier because of immediate transfer to rehabilitation, whereas in Florence, discharge occurred only after completion of rehabilitation. Haemostatic coverage was continued during rehabilitation, although these data are not available.

Haemostatic efficacy during surgery was evaluated intraoperatively based on the clinical judgment of the surgeon on bleeding control and categorized as either ‘good’ or ‘poor.’ Postoperative haemostasis was monitored through clinical and laboratory parameters, including serial haemoglobin (Hb) measurements and transfusion requirements. All adverse events and postoperative complications were systematically recorded. Anaemia was defined according to World Health Organization (WHO) classification in mild (Hb between 10 and 11.9 g/dL), moderate (Hb between 8 and 9.9 g/dL) or severe (Hb below 8 g/dL).

## 3 Results

A total of 20 consecutive major orthopaedic interventions were performed in 19 adult persons with haemophilia A or B. One patient with haemophilia A underwent two procedures on different joints. The cohort consisted of 14 persons with haemophilia A and five with haemophilia B, all male, with a median age of 46 years (range 26–60 years) (Table 1). All participants were treated with a prophylaxis regimen according to the current International Society on Thrombosis and Haemostasis (ISTH) guidelines [7].

**TABLE 1:** Demographic and clinical characteristics.

Age, years, median (range)	46 (26–60)
Weight, kg, median (range)	75 (50–90)
Haemophilia type, *n* (%)	
A moderate	2 (11%)
A severe	12 (63%)
B moderate	0 (0%)
B severe	5 (26%)
Hospitalization, days, median (range)	7 (2–18)
Type of intervention, *n* (%)	
Ankle arthroplasty	9 (45%)
Primary knee arthroplasty	5 (25%)
Revision knee arthroplasty	2 (10%)
Ankle arthroscopic synovectomy	2 (10%)
Ankle arthrodesis	1 (5%)
Femur fracture fixation device removal	1 (5%)

Orthopaedic procedures included ankle arthroplasty (*n* = 9, 45%), primary knee arthroplasty (*n* = 5, 25%), revision knee arthroplasty (*n* = 2, 10%), ankle arthroscopic synovectomy (*n* = 2, 10%), ankle arthrodesis (*n* = 1, 5%), removal of the fixation device due to intolerance after femoral fracture repair (*n* = 1, 5%) (Table 1). Only three procedures were conducted using a tourniquet (all were ankle arthroplasties). Each patient received a preoperative IV. infusion of the deficient recombinant factor following current international recommendations [3].

**FIGURE 1:**
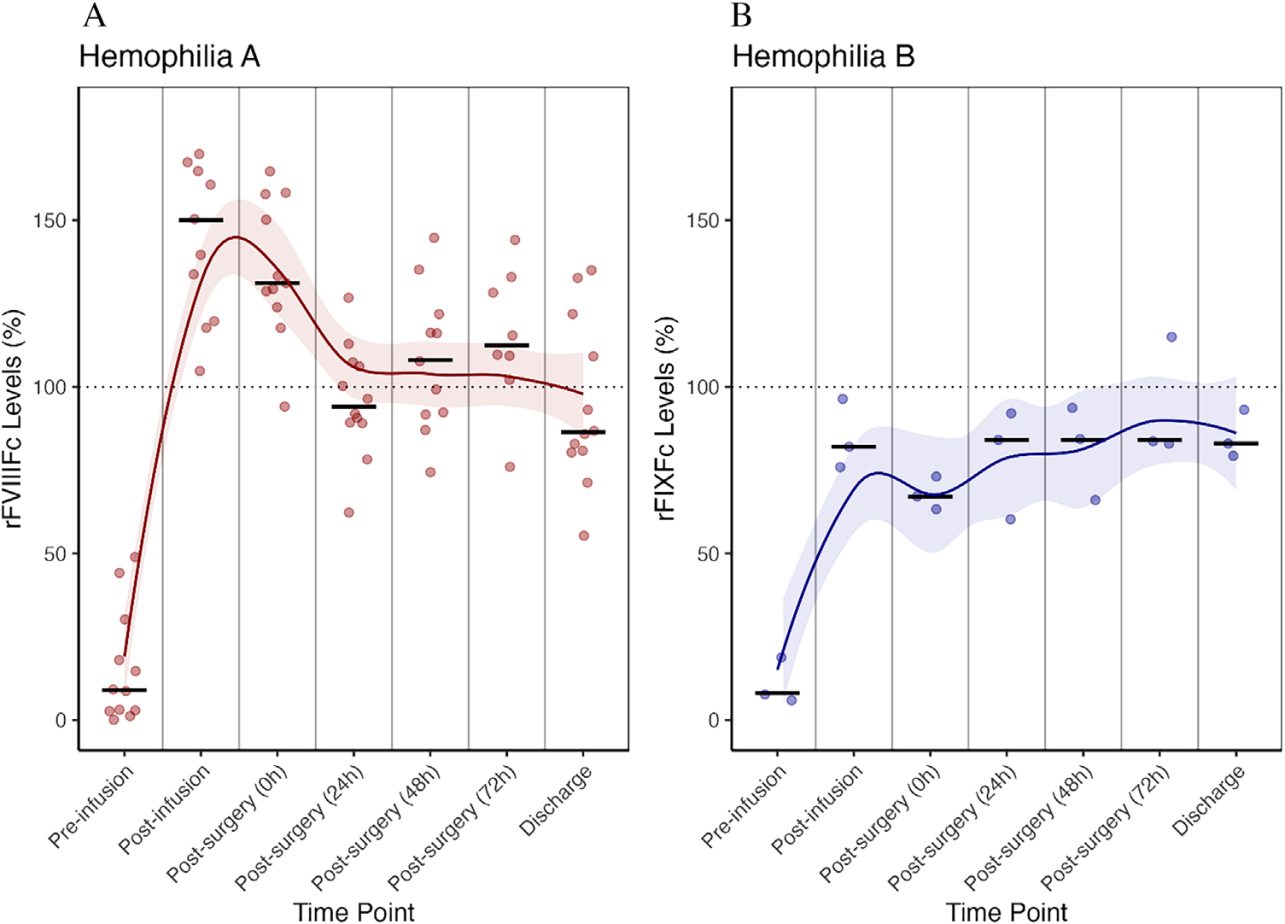
Levels of recombinant factor VIII and IX Fc‐fusion proteins (rFVIIIFc/rFIXFc) during hospitalization. Factor activity levels are expressed as percentages (%), with the target 100% activity level indicated by a dotted line. Data from persons with haemophilia A treated with rFVIIIFc (A) and data from persons with haemophilia B treated with rFIXFc (B). Each scatter dot represents an individual patient's factor level, while horizontal black lines denote the median value for each time point. The coloured lines represent the smoothed trend of factor levels over time, and the shaded areas surrounding the lines indicate the 95% confidence interval (CI) of the trend.

For patients with haemophilia A, the median preoperative FVIII activity level was 12 IU/dL (range 0–49 IU/dL), increasing to 150 IU/dL (range 118–181 IU/dL) following the initial loading dose infusion (Figure 1). Median preoperative factor dose was 60 IU/kg, with total factor dosing on the day of surgery (including postoperative boluses) being 90 IU/kg (range 50–125 IU/kg). Postoperative FVIII levels showed stable haemostatic control, with median factor levels recorded at 129 IU/dL (range 94–165 IU/dL) immediately after surgery, followed by 98 IU/dL (range 62–127 IU/dL) at 24 h, 108 IU/dL (range 74–145 IU/dL) at 48 h, 110 IU/dL (range 76–144 IU/dL) at 72 h and finally 86 IU/dL (range 55–135 IU/dL) at discharge or 1 week postoperatively. The median total factor VIII consumption during hospitalization was 300 IU/kg (range 138–740 IU/kg) (Table 2A).

**TABLE 2:** Surgical outcomes of the studied population. (A) Daily recombinant factor VIII and IX Fc‐fusion proteins (rFVIIIFc/rFIXFc) infusion during hospitalization for haemophilia A and B. Values are expressed in IU/kg. The daily median factor infusions are shown at different time points during hospitalization: day of surgery, 24, 48 and 72 h post‐surgery, and total amount of infused factor at discharge. (B) Summary of intraoperative haemostatic efficacy, postoperative complications, factor‐related adverse events, median haemoglobin change, tranexamic acid administration and treatment duration and use of tourniquet.

A				
	Haemophilia A		Haemophilia B	
Time point	Median	Range [min–max]	Median	Range [min–max]
Day of surgery	90	50–125	92	57–141
24 h post‐surgery	52	30–86	86	29–100
48 h post‐surgery	40	22–80	80	55–88
72 h post‐surgery	31	15–77	62	28–80
Total	300	138–740	388	86–588

B	
Intraoperative haemostasis, *n* (%)	
Good	18 (90%)
Not documented	1 (5%)
Poor	1 (5%)
Complications, *n* (%)	
Anaemia	8 (40%)
Blood loss	1 (5%)
None	11 (55%)
Orthopaedic	0 (0%)
Factor side effects, *n* (%)	
No	20 (100%)
Yes	0 (0%)
Haemoglobin change (g/dL), median (range)	−2 (–4.6 to +0.5)
TXA use, *n* (%)	
No	1 (5%)
Yes	19 (95%)
TXA treatment, days, median (range)	5 (2–14)
Tourniquet use, *n* (%)	
No	17 (85%)
Yes	3 (15%)

Abbreviation: TXA, tranexamic acid.

Patients with haemophilia B had median preoperative factor IX levels of 7 IU/dL (range 2–19 IU/dL), increasing to 82 IU/dL (range 76–96 IU/dL) following the loading dose (Figure 1). Median preoperative dosing was 81 IU/kg, and total dosing on the surgical day was 92 IU/kg (range 57–141 IU/kg) (Table 2A).

Postoperative FIX activity remained stable, with median levels at 65 IU/dL (range 37–75 IU/dL) immediately post‐surgery, 74 IU/dL (range 40–93 IU/dL) at 24 h, 84 IU/dL (range 58–109 IU/dL) at 48 h, 83 IU/dL (range 55–115 IU/dL) at 72 h and 79 IU/dL (range 57–93 IU/dL) at discharge. The median total factor IX consumption during hospitalization was 388 IU/kg (range 86–588 IU/kg) (Table 2A). The median number of deficient factor infusions per patient during hospitalization was 9.5 (range, 5–15) for rFVIIIFc and 11 (range, 7–15) for rFIXFc.

Haemostatic efficacy was rated as ‘good’ in 95% of cases (*n* = 18). One case (5%) was rated as ‘poor’, and data were unavailable for one patient (Table 2B). Median Hb change during hospitalization was –2 g/dL (range: –4.6 to +0.5 g/dL). Anaemia was reported in eight cases (40%), without association with a specific type of intervention, all graded as mild except one case of moderate anaemia in a patient undergoing total knee prosthesis, and no patient required blood transfusions. Tranexamic acid was administered in 95% of the procedures (*n* = 19) for a median duration of 5 days (range 2–14 days).

Complications occurred in 45% (*n* = 9) of patients, primarily anemia in 40% (*n* = 8) and minor surgical site bleeding in 5% of cases (*n* = 1). These complications were managed with oral supplementation of iron and folates. Notably, no thrombotic events were observed during the study period, despite the absence of antithrombotic prophylaxis; only elastic compression stockings and early mobilization were employed, and no adverse reactions to EHL recombinant factor products occurred. The median duration of hospital stay was 7 days (range 2–18 days). No reinterventions, additional procedures or delayed discharges were observed.

## 4 Discussion

In this multicentre prospective study, we describe the surgical outcomes of 20 major orthopaedic procedures of the lower extremities in a cohort of 19 individuals with haemophilia, using EHL replacement therapy during the perioperative period. Our findings demonstrate that replacement with rFVIIIFc and rFIXFc allowed us to effectively achieve and maintain target factor levels with minimal fluctuations throughout the perioperative period, with a reduced bleeding risk and no observed adverse events. Nevertheless, significant variability in factor consumption was noted.

Despite significant advances in the management of individuals with haemophilia, orthopaedic procedures remain a common form of major surgery in this population [8, 9]. Real‐world data are crucial to harmonize clinical practices across centres and evaluate both safety and efficacy of EHL products for major orthopaedic surgery.

Median preoperative doses in our cohort—60 IU/kg for rFVIIIFc and 81 IU/kg for rFIXFc—were comparable to those reported in previous studies [10, 11] and resulted in preoperative factor levels closely approximating the values recommended by the WFH for higher‐dose practice patterns [3]. In line with our study, the median preoperative loading dose of rFVIIIFc in the phase‐3 A‐LONG study was 59 IU/kg for major surgery [12]. On the other hand, in a sub‐analysis of the phase‐3 B‐LONG study, rFIXFc was evaluated for its efficacy in preventing bleeding during 14 major surgeries, 11 of which were orthopaedic procedures. The median preoperative infusion dose in that study was 90.9 IU/kg, slightly higher than the median dose of rFIX administered in the present study. The authors noted that these surgeries represented early experiences with perioperative rFIXFc administration, possibly leading to a cautious and liberal dosing approach based on prior familiarity with conventional SHL FIX products [13]. In the following years, several case series and reports detailed preoperative dosing regimens employed during orthopaedic procedures. In a retrospective case series from the Nordic haemophilia treatment centres conducted in persons with severe haemophilia A undergoing major surgeries [11], median preoperative rFVIIIFc dosing was 48 IU/kg, slightly lower than the dose used in our cohort. This difference may be attributable to the variability in the surgical procedures included in that study. Some of them were minor procedures associated with a lower bleeding risk compared to major orthopaedic interventions, thus requiring a lower preoperative dosage of factor. On the other hand, our Centres are tertiary hub referral Centres for persons with haemophilia that need to undergo more invasive procedures such as total joint replacement. Additionally, a case report from an Italian group in Turin described a moderate haemophilia B patient undergoing hip replacement receiving a preoperative bolus of 80 IU/kg rFIXFc [14], in line with our experience with patients receiving EHL FIX. A collection of phase‐3 studies [15] reported a median loading dose of 59 IU/kg (rFVIIIFc) and 100 IU/kg (rFIXFc) for major surgeries, similar to our experience.

Overall, the reported efficacy rates align with previous studies, reinforcing the reliability of EHL therapies in maintaining stable haemostasis [10, 15, 16]. The high proportion of cases with surgeon‐reported good intraoperative haemostasis further supports the use of these products as a standard option in surgical settings. The single case of poor haemostatic control highlights the need for individualized treatment strategies, particularly for complex or high‐risk patients. In the extended follow‐up of phase‐3 studies (A‐LONG and B‐LONG) including patients undergoing surgeries and receiving EHL factors—ASPIRE [17] and B‐YOND [18]—median estimated blood loss during the perioperative period was 90 mL for haemophilia A and 100 mL for haemophilia B, and the vast majority of cases with blood loss ≥500 mL were orthopaedic surgeries of lower extremities [15]. This is in line with our findings.

Notably, neither thromboembolic events nor inhibitors were observed in our study. These findings are consistent with previous literature, such as the long‐term follow‐up data from pivotal registration trials of both rFVIIIFc and rFIXFc, which have demonstrated the low immunogenicity and good tolerability of these agents [15].

Antifibrinolytic agents are routinely employed in our centres during orthopaedic surgery of individuals with haemophilia [19, 20]. In our cohort, tranexamic acid likely contributed to the effective management of perioperative bleeding, reinforcing its role as a valuable adjunct in haemophilia care.

Our study has some limitations. The variability in factor consumption among persons in our cohort likely reflects differences in baseline factor levels obtained by prophylactic treatment more than haemophilia severity—as both moderate and severe phenotypes were included. This warrants personalized dosing protocols that account for patient‐specific factors such as baseline factor levels, surgical complexity and individual pharmacokinetics. Another limitation is that factor levels during the rehabilitation period were not available; however, factor coverage was maintained, and data from the first 48–72 h, when perioperative bleeding risk is highest, are generally considered the most clinically relevant [3]. The limited sample size—comprising 20 surgical interventions, only five of which were in individuals with haemophilia B (including one patient who underwent two procedures)—did not allow for the establishment of correlations between specific interventions and bleeding risk. However, both Centres serve as national referral hubs with extensive experience in surgical management and comprehensive care of individuals with haemophilia—a rare disorder in which the number of patients undergoing surgical procedures is inherently limited. In this context, the sample size cannot be considered particularly small.

Another key consideration is the cost‐effectiveness of EHL therapies compared to SHL products. Despite higher upfront costs of EHL factors, significant benefits in reducing infusion frequency may lead to long‐term cost‐effectiveness [21]. Future research should focus on cost‐benefit analyses to guide healthcare decision‐making and ensure equitable access to these advanced therapies.

## 5 Conclusion

In conclusion, our study supports the role of rFVIIIFc and rFIXFc as effective and safe options for managing haemostasis in major orthopaedic surgery for persons with haemophilia.

Importantly, maintaining factor levels above 100 IU/dL during the early postoperative period, in particular during the first 48–72 h, was associated with satisfactory bleeding control in patients with haemophilia A. Although median FIX trough levels did not exceed 100 IU/dL, they remained sufficiently high to prevent bleeding episodes in patients with haemophilia B as well, with no need for transfusions in either group. Further multicentre studies are warranted to confirm these results and refine perioperative management guidelines.

## Ethics Statement

The study was approved by the Ethics Committee of Fondazione IRCCS Ca’ Granda Ospedale Maggiore Policlinico in Milan and was conducted in conformity with the 2013 revision of the Declaration of Helsinki and the code of Good Clinical Practice.

## Conflicts of Interest

Luigi Piero Solimeno: Sobi, Biomarin (grants). Roberta Gualtierotti: Bayer, Biomarin, Roche, Sanofi, Sobi, Pfizer, Takeda and Novo Nordisk (advisory board/educational meetings or symposia). Flora Peyvandi: Biomarin, CSL Behring, Pfizer, Roche, Sanofi, Sobi, Takeda (advisory board/educational meetings or symposia). All other authors stated that they had no interests which might be perceived as posing a conflict or bias.

## Data Availability

The data that support the findings of this study are available on request from the corresponding author. The data are not publicly available due to privacy or ethical restrictions.
